# Dismal outcome if delayed cardiac surgery because of coronavirus disease 2019

**DOI:** 10.1093/icvts/ivac072

**Published:** 2022-03-15

**Authors:** Torbjörn Ivert, Magnus Dalén

**Affiliations:** 1 Department of Cardiothoracic Surgery, Karolinska University Hospital, Stockholm, Sweden; 2 Department of Molecular Medicine and Surgery, Karolinska Institutet, Stockholm, Sweden

**Keywords:** Coronavirus disease 2019, Cardiac surgery, Delayed treatment

## Abstract

The coronavirus disease 2019 (COVID-19) pandemic was a great burden for health care worldwide. We encountered 21 non-infected adult patients during 2020 who deferred to seek medical treatment since they thought that their difficulties to breathe were due to COVID-19. They were diagnosed late with cardiac disease with the indication for surgery. Deferred surgery for aortic stenosis was the cause of death in 1 patient. Long-standing not-treated endocarditis had caused severe aortic root pathology in 3 patients. Late-diagnosed ST-elevation myocardial infarction in 2 patients had caused papillary muscle and ventricular wall rupture. Eighteen of the patients finally underwent heart surgery at our tertiary care centre with early mortality of 22%. We conclude that late diagnosis of subjects requiring surgical treatment for heart disease was a risk for dismal outcomes during the COVID-19 pandemic.

## INTRODUCTION

Severe acute respiratory syndrome coronavirus 2 causes an infectious syndrome [coronavirus disease 2019 (COVID-19)] sometimes manifested by severe acute hypoxic respiratory failure that may require long hospitalization in vulnerable individuals with often fatal outcomes [1]. Severity ranges from a spectrum of almost no symptoms to respiratory failure with circulatory collapse, cytokine storm and death [2]. Sweden was hit by 4 waves of infection. The last small wave faded out during the fall of 2021. The maximal incidence of cases occurred during 2020. Massive vaccination was started during the spring of 2021. Citizens were advised to restrict measures, avoid public communication, keep distance to other subjects and, if possible, work from home during the pandemic to reduce risks of transmission in the community. Subjects with the onset of fever and difficulties breathing sometimes assumed that their symptoms were caused by COVID-19 and were reluctant to seek medical treatment. This delayed the diagnosis of severe cardiac illness in some subjects.

Our aim was to analyse the consequences of delayed diagnosis and management in candidates for cardiac surgery during the pandemic and evaluate the patient outcome.

## PATIENTS AND METHODS

During 2020, we encountered 21 non-infected adult patients who thought that they had COVID-19 and were admitted because of difficulties breathing and often cough, fatigue and fever. Typically, some patients who had lived in isolation for several months due to the fear of acquiring COVID-19 infection had asked for testing several times. One patient had been tested 6 times. All COVID-19 tests were negative. Assessment of correct diagnosis start of medication and cardiac evaluation were delayed. They were finally referred for cardiac surgery at Karolinska University Hospital, Stockholm, Sweden (Table 1). There were 6 women (29%) and age ranged from 28 to 79 years. They were diagnosed with endocarditis (*n* = 6), aortic (*n* = 6) or mitral valve (*n* = 4) dysfunction, coronary artery disease (*n* = 3) or myocardial infarction (*n* = 2). Five patients had previous cardiac surgery. In 2 subjects, emergency room evaluation was delayed because of waiting for COVID-19 test results (cases 1 and 8). During the pandemic, patients admitted emergently with airways symptoms were regarded as probable COVID-19 patients and were initially isolated. Further evaluations were postponed until negative COVID-19 test was obtained usually within 24 h. This study was approved by the Swedish Ethical Review Authority (Dnr. 2020-05209).

**Table 1:** Delayed management because of coronavirus disease 2019 in 21 patients admitted for heart surgery during 2020

Case	Diagnosis	Age	Sex	Cause	Delay	Procedure and outcome	Died
1	Endocarditis	81	M	Infected composite graft. Bleeding caused cardiac tamponade	5 days	Death on admittance, emergency surgery planned	Yes
2	Endocarditis	70	F	Vegetations on aortic bioprosthesis	2 months	Healed after medical treatment	No
3	Endocarditis	63	M	Aortic and mitral valve endocarditis	3 months	Aortic and mitral valve replacement, uneventful recovery	No
4	Endocarditis	45	F	Native aortic valve endocarditis.	1 months	Root replacement, uneventful recovery	No
5	Endocarditis	43	F	Aortic and mitral valve endocarditis	2 months	Root replacement, mechanical aortic and mitral valves. Heart failure, ECMO, pacemaker, dialysis, recovered	No
				Left ventricular failure.			
6	Endocarditis	28	M	Tricuspid valve endocarditis	3 months	Tricuspid valve replacement, uneventful recovery	No
7	Aortic valve	74	F	Degenerated aortic bioprosthesis	9 months	Transcatheter aortic valve-in-valve implantation, uneventful	No
8	Aortic valve	71	M	Degenerated aortic bioprosthesis	5 days	Redo aortic valve replacement, uneventful	No
9	Aortic valve	59	M	Aortic stenosis	3 months	Death while awaiting surgery	Yes
10	Aortic valve	56	M	Aortic valve insufficiency	3 months	Aortic valve replacement, uneventful	No
11	Aortic valve	51	F	Aortic and mitral valve insufficiency	5 months	Aortic valve replacement, mitral valve repair, uneventful	No
12	Aortic valve	52	M	Aortic valve insufficiency	12 months	Aortic valve replacement, uneventful	No
13	Mitral valve	77	M	Heart failure, mitral valve insufficiency	5 months	Mitral valve replacement, uneventful	No
14	Mitral valve	75	M	Heart failure, mitral valve insufficiency	10 months	Mitral valve repair, uneventful	No
15	Mitral valve	54	M	Mitral valve insufficiency	6 weeks	Mitral valve replacement, uneventful	No
16	Mitral valve	48	M	Mitral valve insufficiency	3 weeks	Mitral valve repair later valve replacement, uneventful	No
17	CAD	73	M	Acute coronary syndrome	2 months	Coronary artery bypass, uneventful	No
18	CAD	65	M	Stable angina	5 months	Coronary artery bypass, uneventful	No
19	CAD	49	F	Heart failure	7 months	Coronary stenting because of poor left ventricular function	No
20	AMI	71	M	Left ventricular rupture	25 days	Death in the operating room.	Yes
21	AMI	79	M	Papillary muscle rupture	10 days	Death 7 days after mitral valve replacement	Yes

AMI: acute myocardial infarction; CAD: coronary artery disease; ECMO: extracorporeal membrane oxygenation; F: female; M: men.

## RESULTS

Three of the 21 patients did not undergo open cardiac surgery. A 70-year-old woman with cusp vegetations on an aortic bioprosthesis and *Streptocoocus mitis* in blood cultures was cured after antibiotic treatment, and surgery was not required (case 2). A 74-year-old man with a degenerated obstructive aortic bioprosthesis was treated with transcatheter aortic valve-in-valve implantation (case 7). A 49-year-old woman with coronary artery obstruction unsuitable for bypass grafting and poor left ventricular function had chronic total occlusion percutaneous coronary intervention with stenting (case 19).

There were 4 early deaths (22%) among the 18 surgically treated patients. One patient with recurrent aortic root infection died suddenly from cardiac tamponade during transportation before planned surgery. A 59-year-old man with three-vessel coronary artery disease and aortic stenosis died while waiting for surgery that was deferred because of the pandemic (case 9) and 2 patients died from mechanical complications of late-diagnosed ST-elevation myocardial infarction (cases 20 and 21).

Five patients with endocarditis initially thought that they had COVID-19. In 3 patients, long-standing streptococci infection caused advanced aortic root pathology so that double valve replacement or root replacement was necessary (cases 3–5). In a 28-year-old man with a longstanding right-sided infection, the tricuspid valve had to be replaced by a bioprosthesis (case 6).

At follow-up in October 2021, there were no further deaths, no recurrent infection and all survivors had double COVID-19 vaccination.

## DISCUSSION

Very delayed cardiac surgery because of COVID-19 was rare in our hospital and the group constituted only 1.8% of all cases operated on during 2020 (18/1024). The outcome was poor in the operated group with 22% early deaths, compared with all other patients operated at our centre in 2020 with 0.7% early deaths (7/1006). Particularly, late diagnosis of acute myocardial infarction was associated with papillary muscle and left ventricular free wall rupture. Hazard of late diagnosis of ST-segment myocardial infarction during the coronavirus pandemic associated with ventricular septal defect and papillary muscle rupture has been reported by Alsidawi *et al.* [3]. Patients with symptomatic aortic stenosis should be managed without unnecessary delay [4]. We experienced that late diagnosis and start of antibiotic treatment in case of endocarditis had caused extensive aortic root pathology.

Our report highlights that dismal outcomes may follow when the diagnosis and treatment of severe cardiac disease is delayed [5]. During the pandemic, non-specific COVID-19 symptoms such as fever, cough and dyspnoea might have overshadowed other possible diagnoses and caused misdiagnosis with adverse outcomes for patients [1].

### Limitations

We included in this analysis only patients referred for cardiac surgery during 2020 in whom it was documented in the patient charts that COVID-19 was the reason for the delay of treatment.

## CONCLUSION

We conclude that late diagnosis of subjects requiring surgical treatment for heart disease was a risk for dismal outcomes during the COVID-19 pandemic.

## Funding

Magnus Dalén was supported by a donation from Fredrik Lundberg.

**Conflict of interest:** none declared.

## Reviewer information

Interactive CardioVascular and Thoracic Surgery thanks the anonymous reviewer(s) for their contribution to the peer review process of this article.
